# Further Success with Nitre as a Remedy in Chill and Fever

**Published:** 1890-03

**Authors:** J. D. Hunter

**Affiliations:** New Orleans, La.


### FURTHER SUCCESS WITH NITRE AS A REMEDY IN CHILL AND FEVER.

NEW ORLEANS, LA., Feb. 17, 1890.

*Editor Daniel's Texas Medical Journal:*

Since I placed in your hands for publication my brief paper on potassii nitras, I have concluded to add my experience in its use for the past few days, which will be suggestive as to the manner of employing the salt.

I was called, on February 4th, to see a lady from the country, who, for several months, suffered from chill and fever; chill occurred on alternate days. Condition intensely jaundiced, considerably emaciated, entire loss of appetite, exhausted by the least exertion. Ordered thirty grain doses of potassii nitras to be given two hours before the time of chill, and another similar dose when symptoms of chill occurred. No chill took place. The same doses ordered on the next alternate day; no recurrence of chill, and no further medication employed. Jaundice cleared up completely, appetite restored, convalescence established. Patient discharged February 12th.

Was called on Wednesday to see a young lady of the city, who was seized with chill and fever the preceding Sunday. Chills occurred at 6 o'clock, every evening. Gave quinine sulphas for two days, with no beneficial result. Ordered thirty grain doses of potassii nitras, to be given as in the case above detailed. No chill took place on the first day; on the second day, at the hour of six, the usual symptoms of chill were felt. A thirty grain dose of the salt was at once given, with the effect of immediate abortion. No further medication; patient is now attending to her usual household duties.

I visited, on Sunday at two o'clock, a railroad engineer, who was attacked with chill and fever, in the State of Mississippi, the preceding Thursday. Chill occurred daily about twelve o'clock, continuing one hour, followed by fever which lasted eight hours. Found patient with high fever; temperature, 104° S. L. Gave ten grains antifebrin; profuse perspiration and lowering of temperature to 99° followed. Fever rose again at four o'clock; temperature at eight o'clock, 103°. Fever continued as heretofore until ten o'clock. Ordered forty-five grains of potassii nitras to be given at ten o'clock next day, and a similar dose when symptoms of chill were felt. No chill took place. At ten o'clock the succeeding day forty-five grains of the salt was again given, with instructions to take a similar dose on occurrence of chill; no chill occurred. Patient was permitted to resume his occupation on Thursday, with instructions to report. In two other cases (also railroad men), identical with this, the paroxysms were shortened but a cure was not effected.

A gentleman from New York contracted chill and fever while on the Isthmus of Panama. Failing to obtain relief, he came to New Orleans for treatment. Chill occurred on alternate days, followed by fever of ten hours duration, and exhausting sweats. Found relief from quinine and arsenic; relapses were frequent. So confident was I of success that I promised a speedy cure. Gave thirty grains pottassii nitras two hours preceding chill; another dose when chill occurred. In this case no beneficial result followed. The chill, fever and sweat were of the usual intensity and duration. Sixty grain doses, given in the same manner on the next alternate day, resulted in a like failure. Interrupted the chill with quinine sulphate and oil of black pepper, in large doses. Patient is now upon this treatment.

Chill and fever is of unusual occurrence in New Orleans, consequently the field for observation is limited. The majority of cases treated by me were from the country and were of long standing. At least four-fifths of the cures effected were in cases of a chronic character, which did not yield to quinine or arsenic. Failures may be expected in recent cases.

To-day a young man called at my offce, suffering from a small gluteal abscess and a fissure of anus. Upon opening the abscess and cauterizing the fissure, he was seized with a violent chill. Administered spirits of ammonia and brandy. Condition unchanged after a lapse of fifteen minutes; temperature 96° S. L. Surface cold and clammy, teeth chattering, face blue and pinched. I thought of potassii nitras, and concluded to hazard a dose. Gave thirty grains. Within seven minutes chill ceased, temperature normal, circulation re-established. Within ten minutes from the time of administering the salt, the patient was on the street walking home. This is to me a new experience. Heretofore I employed the salt only in chills presumably malarial. To abort a malarial chill has heretofore been difficult, nay, I may say impossible of accomplishment. To abort and at the same time to effect a radical cure approximating uniformity with a few grains of a simple salt, so far as I know, is without precedent in medical experience.

Very respectfully yours,

J. D. HUNTER, M. D.

